# Lipid Driven Nanodomains in Giant Lipid Vesicles are Fluid and Disordered

**DOI:** 10.1038/s41598-017-05539-y

**Published:** 2017-07-14

**Authors:** Alena Koukalová, Mariana Amaro, Gokcan Aydogan, Gerhard Gröbner, Philip T. F. Williamson, Ilya Mikhalyov, Martin Hof, Radek Šachl

**Affiliations:** 1 Department of Biophysical Chemistry, J. Heyrovský Institute of Physical Chemistry of the A.S.C.R. v.v.i., Prague, Czech Republic; 2 Department of Chemistry, University of Umeå, SE-901 87 Umeå, Sweden; 3 Centre for Biological Sciences/Institute for Life Sciences, University of Southampton, Southampton, SO17 1BJ United Kingdom; 4 Shemyakin-Ovchinnikov Institute of Bioorganic Chemistry of the Russian Academy of Science, Moscow, GSP-7 Russian Federation

## Abstract

It is a fundamental question in cell biology and biophysics whether sphingomyelin (SM)- and cholesterol (Chol)- driven nanodomains exist in living cells and in model membranes. Biophysical studies on model membranes revealed SM and Chol driven micrometer-sized liquid-ordered domains. Although the existence of such microdomains has not been proven for the plasma membrane, such lipid mixtures have been often used as a model system for ‘rafts’. On the other hand, recent super resolution and single molecule results indicate that the plasma membrane might organize into nanocompartments. However, due to the limited resolution of those techniques their unambiguous characterization is still missing. In this work, a novel combination of Förster resonance energy transfer and Monte Carlo simulations (MC-FRET) identifies directly 10 nm large nanodomains in liquid-disordered model membranes composed of lipid mixtures containing SM and Chol. Combining MC-FRET with solid-state wide-line and high resolution magic angle spinning NMR as well as with fluorescence correlation spectroscopy we demonstrate that these nanodomains containing hundreds of lipid molecules are fluid and disordered. In terms of their size, fluidity, order and lifetime these nanodomains may represent a relevant model system for cellular membranes and are closely related to nanocompartments suggested to exist in cellular membranes.

## Introduction

The original definition of rafts as sphingomyelin (SM)- and cholesterol (Chol)-enriched platforms in cellular plasma membranes emerged over 20 years ago^1^. The postulation of such heterogeneities found support in experiments where detergent resistant membranes biochemically isolated from cells were shown to be enriched in SM and Chol^1, 2^. This was supported by biophysical studies on model membranes (i.e. giant unilamellar vesicles; GUVs) composed of SM, Chol and phosphatidylcholine which revealed micrometer-sized liquid-ordered (L_o_) domains. Recently, another valuable model membrane system was developed and characterised. The so-called giant plasma membrane vesicles (GPMVs) are formed directly from cells and thus the formed membrane contains multiple essential components of the cellular plasma membrane^3^. Interestingly, fluorescence experiments performed on those GPMVs indicated that physicochemical properties of the cell-derived membranes differ significantly from those with synthetic lipid composition^4, 5^. The micrometer-sized ordered phase of GPMVs seems less ordered than L_o_ phase of GUVs and in analogy to that the disordered phase of cell-derived membranes is more ordered than L_d_ phase of GUVs^6^. This suggests that differences between domain and non-domain parts of biological membranes are rather subtle and not so extreme as between L_o_ and L_d_ phase of GUVs formed from synthetic lipids.

The search for L_o_ phase domains in cellular plasma membranes was brought by an attempt to draw analogies between the SM and Chol enriched microdomains and the postulated plasma membrane rafts. Experiments using super-resolution fluorescence imaging techniques^7, 8^ or indirect approaches using polarity sensitive probes^9–11^ suggest that heterogeneity of cellular plasma membranes exists. However, those heterogeneities seem to occur on the nano- rather than on the micro-scale^7, 9, 12, 13^. The failure to directly detect domains in cellular membranes by super-resolution fluorescence imaging techniques with a resolution of about 40 nm^7, 8^ and the recent results concerning properties of biological membranes question the biological relevance of the L_o_ phase microdomains found in model membranes. The assumption that experiments on model membranes can reveal biologically relevant information leads us to two central questions: firstly, can lipid driven domains in model membranes be smaller than 40 nm and secondly if so, do such nanodomains have a L_o_ character?

In this work we used MC-FRET in combination with novel monosialoganglioside GM_1_ fluorescent probes to uncover the existence of nanodomains in lipid bilayers that should be in a homogeneous liquid disordered (L_d_) phase according to published phase diagrams^14, 15^. FRET has been frequently used in the past to reveal micro- to nano-scale heterogeneities in lipid membranes^16–18^ but mostly on a qualitative level. Combination of FRET with MC simulations enabled us quantifying the sizes of domains down to a few nanometers and the fractional area occupied by these domains. To assess the fluidity and phase of the nanodomains we employed solid state wide-line and high resolution MAS (magic angle spinning) NMR spectroscopy, two-color z-scan fluorescence correlation spectroscopy (FCS)^19^ and FRET.

### Determination of nanodomain size and fractional bilayer area by MC-FRET

#### Description of the MC-FRET approach

**Figure 1:**
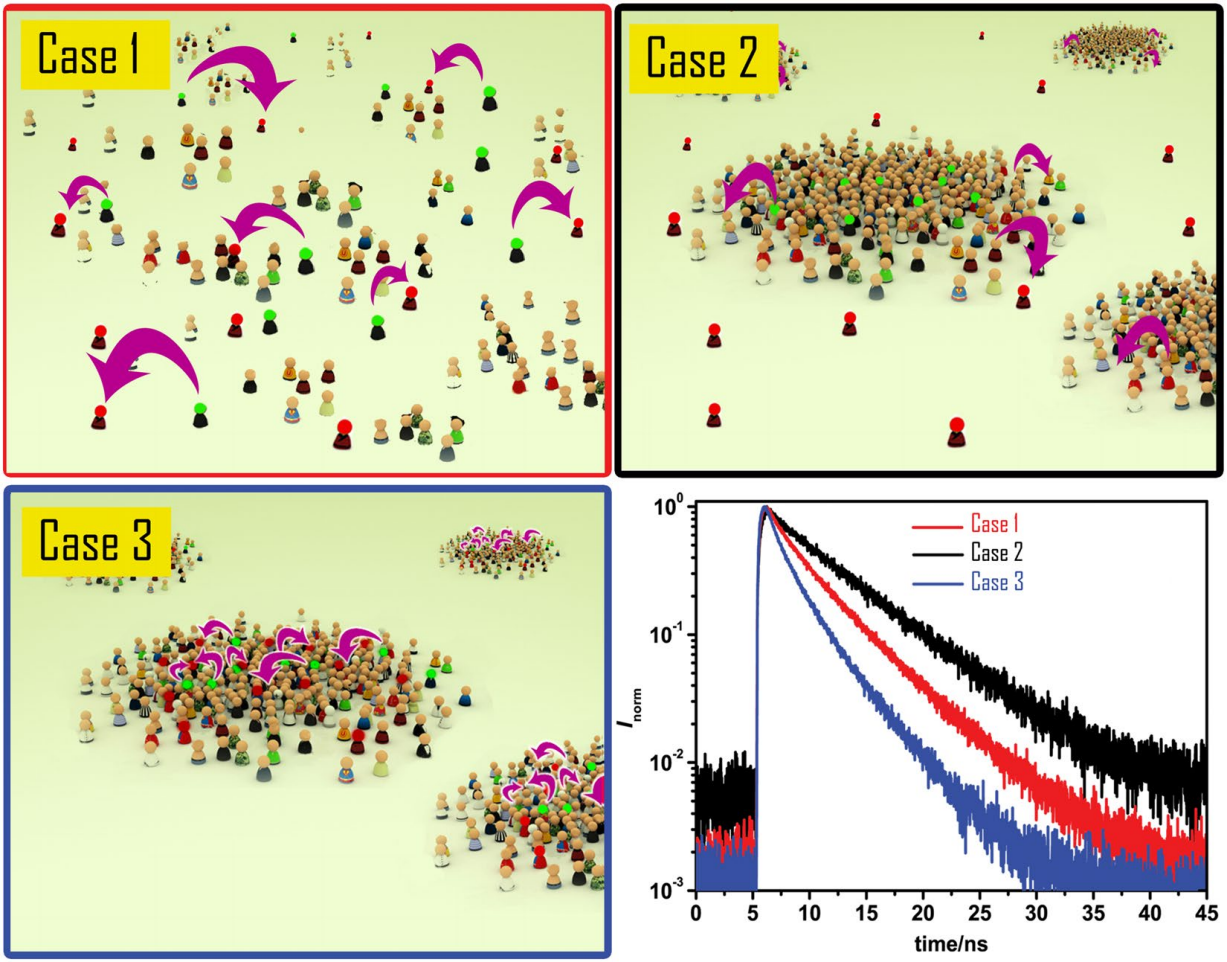
Figure depicting the basic principles underlining the detection of domains by FRET. When donors (green) and acceptors (red) are homogeneously distributed on a plane at sufficiently high acceptor concentration (Case 1) FRET occurs and speeds up the relaxation of the donors back to the ground state. If formation of domains influences the distribution of probes, i.e. when the probes possess different affinity for the domains and the remaining part of the bilayer, two scenarios are possible: Case 2 where the donors accumulate in the domains and acceptors are excluded from them leading to separation of the donors from the acceptors and thus to a decrease in the FRET efficiency and consequently to slower relaxation to the ground state (compare the black with the red decay in the bottom right corner); Case 3 where the accumulation of the donors and acceptors in the nanodomains results in more efficient FRET and consequently to faster relaxation to the ground state (compare the blue with the red decay in the bottom right corner). Bottom right: Examples of the fluorescence decays of the donors in each case. The decays are influenced by the size of the domains, the fractional area occupied by the domains and by the affinity of the probes for the domains; the readouts which can be determined by fitting the experimental decays by using MC-FRET method.

FRET between a single donor and a single acceptor occurs at distances between 1 to 10 nm and can be used as a molecular ruler within this accessible range. The situation is different when FRET occurs in a lipid bilayer that contains nanodomains and an ensemble of heterogeneously distributed donors and acceptors. Here, the formation of nanodomain structures forces a homogeneous distribution of donors and acceptors (Fig. 1, case 1) into a heterogeneous one (Fig. 1, cases 2 and 3) when using appropriate fluorescent probes that possess either an increased or decreased affinity for such nanodomains. This causes a change in FRET efficiency that can be seen in the recorded fluorescence decays (Fig. 1, bottom right corner). In these cases, the range of accessible distances (domain radii) that can be determined is significantly broader (2–50 nm)^20^ and lies exactly in the region where other techniques become less efficient. The remarkably broad range of accessible distances is a consequence of FRET that occurs at the boundary of the nanodomains and of the fact that the length of that boundary depends on the nanodomain radius *R*_D_. The entire process of energy transfer can be modeled using MC simulations under certain assumptions (see Materials and Methods for details). The simulated decay curves were fitted to the experimental data by varying the radius of the nanodomains *R*_D_, the fractional bilayer area occupied by the nanodomains *Ar* (which is proportional to nanodomain concentration *c*_D_ by *c*_D_ = *Ar*/(π*R*_D_)) and the distribution constants of donors *K*_D_(D) and acceptors *K*_D_(A) (defined as *K*_D_(D) = [D_inside_]/[D_outside_], *K*_D_(A) = [A_inside_]/[A_outside_]).

MC-FRET can detect various kinds of membrane heterogeneities, such as domains or pores^21, 22^. However, its resolution significantly depends on both *K*_D_(D) and *K*_D_(A). If the probes possess equal affinity for the nanodomains and the remaining bilayer, then the formation of heterogeneities in a bilayer will not induce a heterogeneous probe distribution and therefore no change in FRET efficiency will occur. Consequently, such heterogeneities will not be ‘seen’ by FRET and the selected probes. Thus, Donor/Acceptor (D/A) pairs with suitable *K*_D_ have to be chosen. Based on literature^23^ and our previous work^24, 25^ we used two different D/A pairs for the detection of lipid driven nanodomains. The first one consisted of ganglioside GM_1_ molecules labeled at the headgroup with either FL-BODIPY (g-GM_1_) or 564/570-BODIPY (r-GM_1_). Both g-GM_1_ and r-GM_1_ show increased affinity for the L_o_ microdomains but also to less ordered fluid nanodomains (refs 24 and 25 and as shown on Table 1 and Table 2). Importantly, these GM_1_ probes do not intrinsically self-aggregate at the concentrations used in the FRET experiments (see SI and ref. 26). The second D/A pair consisted of 1,2-distearoyl-sn-glycero-3-phosphoethanolamine-N-[amino (Polyethyleneglycol) 2000] labeled at the end of the Polyethyleneglycol chain with either carboxyfluorescein (CF-PEG-DSPE) or Rhodamine101 (Rh-PEG-DSPE). PEG-DSPE lipids were shown to have increased affinity for the L_o_ phase^23^, which is confirmed by this work (Table 2). In addition, here we demonstrate that these probes preferentially partition to the nanodomains that are rich in Chol and SM but still maintain their liquid-disordered character.

**Table 1:** The average radius and fractional bilayer area of the nanodomains, distribution constants *K*_D_ and relative FRET efficiencies *E*_rel_ (for definition see Materials and Method section) for two different donor-acceptor pairs in DOPC/SM and DOPC/Chol/SM mixtures.

DOPC (mol%)	SM (mol%)	Chol (mol%)	Domain radius (nm)	Domain area (%)	FRET pair	*K*_D_(D)**	*K*_D_(A)**	*E*_rel_***
100, 75, 70	0	0, 25, 30	Homogeneous distrib.		g-GM_1_/r-GM_1_	-----*	-----*	1.00
95, 92	5, 8	0	Homogeneous distrib.		g-GM_1_/r-GM_1_	-----*	-----*	1.00
90, 88, 85	10, 12, 15	0	8 ± 1	37 ± 10	g-GM_1_/r-GM_1_	≈10	≈10	1.03
			12 ± 3	55 ± 10				
95, 92, 90, 88	5, 8, 10, 12	0	Homogeneous distrib.		CF-PEG-DSPE/Rh-PEG-DSPE	≈1	≈1	1.00
70, 67, 65	5, 8, 10	25	9 ± 1	45 ± 5	g-GM_1_/r-GM_1_	≥20	≥20	1.12
63	12	25	Homogeneous distrib.		g-GM_1_/r-GM_1_	-----*	-----*	1.00
70, 67	5, 8	25	Homogeneous distrib.		CF-PEG-DSPE/Rh-PEG-DSPE	≈1	≈1	1.00
65, 63	10, 12	25	8 ± 1	55 ± 5	CF-PEG-DSPE/Rh-PEG-DSPE	≈5	≈5	1.10
60	10	30	9 ± 1	45 ± 5	g-GM_1_/r-GM_1_	≥20	≥20	1.12

All lipid mixtures that are given in the same row provided overlapping fluorescence decays. For this reason, the same values are determined for these parameters in the mentioned bilayers. The output parameters were determined by MC-FRET. The total amount of D/A molecules was 1 mol% at max. ^*^no nanodomains detected at the given bilayer compositions; ^**^as determined by MC-FRET; ^***^the estimated error in Erel was below 1%.

**Table 2:** Distribution constants *K*_D_ of probes for L_o_ microdomains and implications for FRET in the presence of L_d_ or L_o_ domains.

Donor	*K*_D_ for L_o_ micro domains^*^	Acceptor	*K*_D_ for L_o_ micro domains^*^	Implications for FRET in the presence of L_d_ nanodomains	Implications for FRET in the presence of L_o_ nanodomains
g-GM_1_	2.5 ± 0.28	r-GM_1_	1.6 ± 0.63	Increased^**,***^	Slightly increased^**,***^
CF-PEG-DSPE	1.5 ± 0.54	Rh-PEG-DSPE	3.5 ± 0.90	Slightly increased^**,***^	Slightly increased^**,***^
g-GM_1_	2.5 ± 0.28	DiD	0.1 ± 0.01	No change^**^	Decreased^**^
Atto 488-DOPE	0.29 ± 0.115	Atto 633-DOPE	0.03 ± 0.013	No change^**^	Increased^**^

Note that *K*_D_ for L_d_ nanodomains are shown in Table 1. *Determined by intensity measurements (see SI) in L_d_/L_o_ phase separated bilayers of DOPC/Chol/SM (55/25/20); ^**^as compared to FRET obtained in a homogeneous bilayer; ^***^this conclusion is drawn based on *K*_D_s given in Table 1.

**Figure 2:**
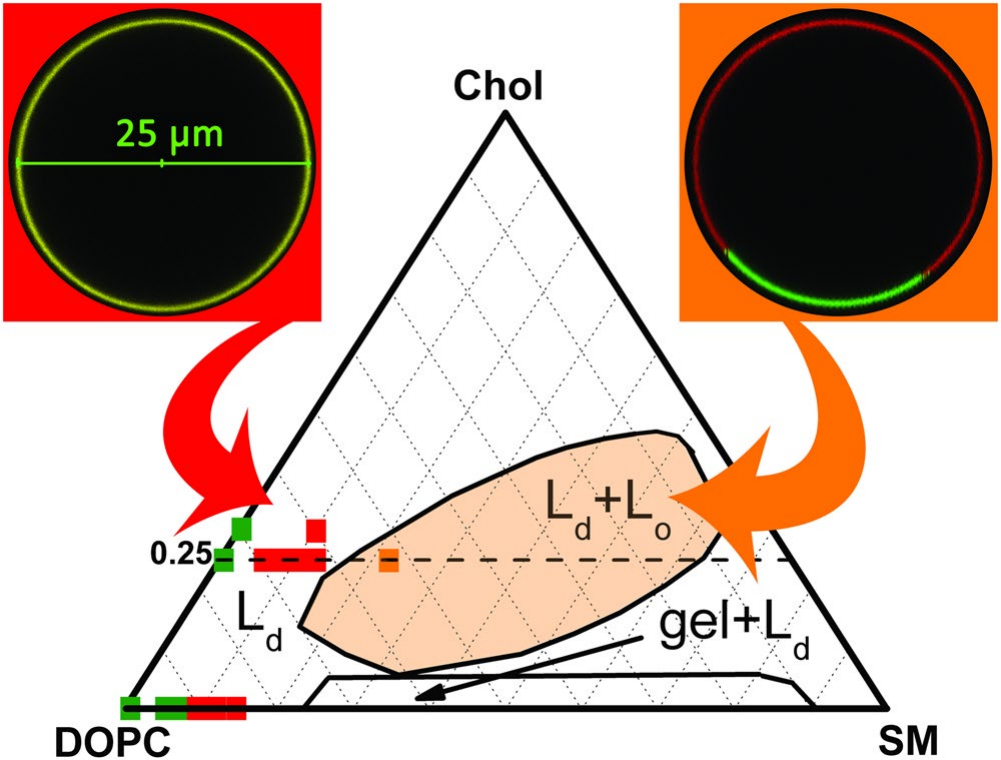
The DOPC/Chol/SM ternary phase diagram. The boundaries for the L_d_/L_o_ and gel/L_d_ regions of phase coexistence were taken from refs 14 and 15. Selected points mark the compositions at which homogeneous bilayers (green squares), bilayers with liquid-disordered (L_d_) nanodomains (red squares) or with microscopic liquid-ordered (L_o_) phase domains (orange square) were found. The fluorescent microscopy images at the top show the apparent homogeneous nature of the bilayers containing nanodomains and the microscopic heterogeneity of mixtures in the L_d_/L_o_ phase coexistence region. The images were obtained by using g-GM_1_ (green) and DiD (red) probes. The details concerning microscope setup are described in the Materials and Method section.

**Figure 3:**
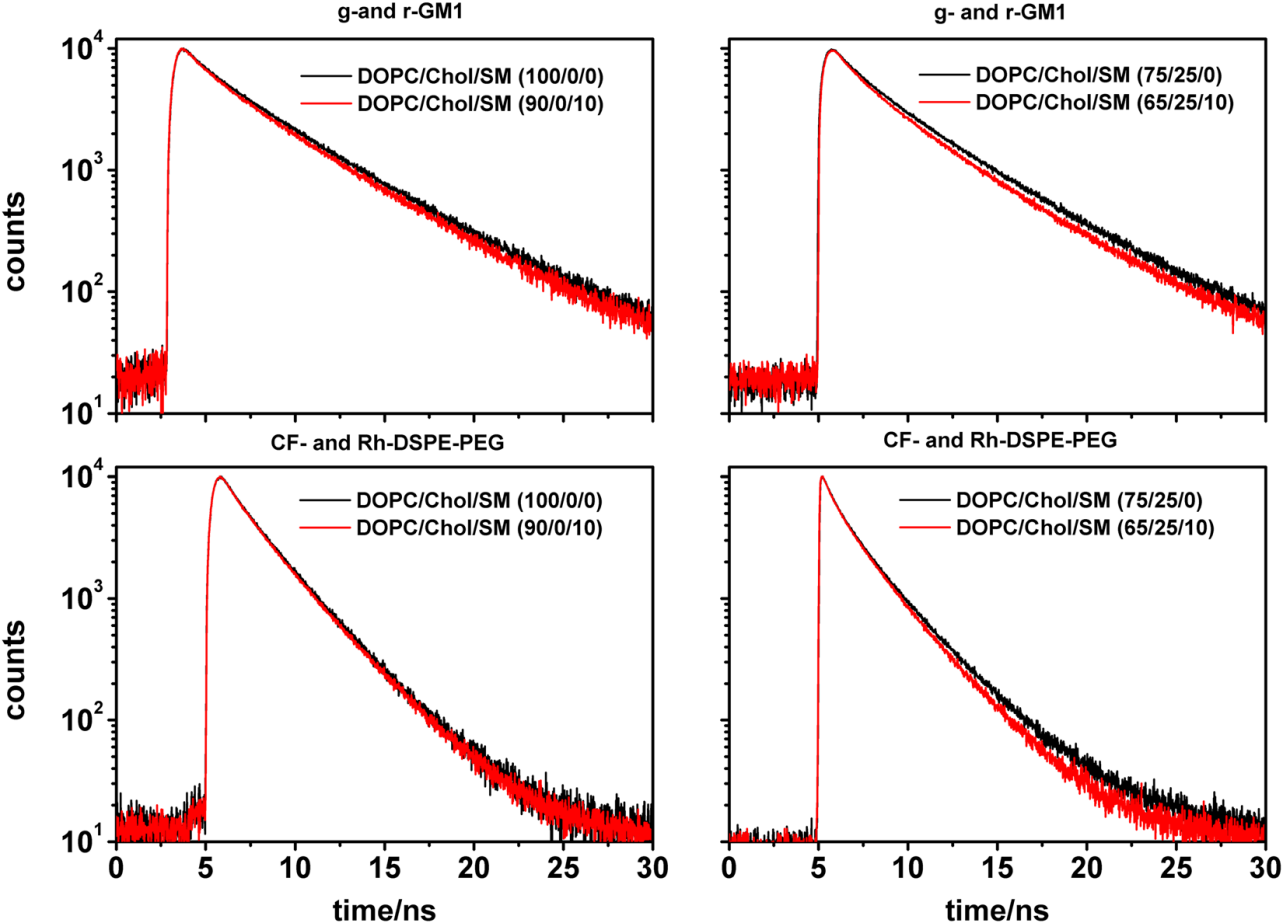
Experimental time-resolved fluorescence decays of the donor for the two D/A pairs and selected lipid mixtures with and without nanodomains (red and black fluorescence decays, respectively).

#### Nanodomains outside the L_*d*_/gel phase coexistence region in DOPC/SM

These mixtures phase-separate at 23 mol% of SM (L_d_ + gel phase coexistence) and are fully converted into the gel phase at 81 mol% of SM at room temperature (Fig. 2)^27, 28^. To detect and characterize nanodomains in this binary system we performed MC-FRET experiments in the range 0–15 mol% of SM with g-GM_1_/r-GM_1_ and CF-PEG-DSPE/Rh-PEG-DSPE D/A pairs. All bilayers appeared homogeneous in confocal images (see an example in Fig. 2). MC-FRET results obtained using g-GM_1_/r-GM_1_ D/A pair indicated that the bilayers were homogeneous at DOPC/SM (100–92/0–8), while at (90–85/10–15) we were able to detect nanodomains (Table 1). The presence of nanodomains is reflected in an enhanced relative FRET efficiency (see Table 1 for results and Materials and Methods for definition) and a faster fluorescence decay of the donor g-GM_1_ in the presence of r-GM_1_ (compare the two decays in the top left panel of Fig. 3). Determination of average domain radius yielded two global minima at *R*_D_ = (8 ± 1) nm, *Ar* = (37 ± 10) % and *R*_D_ = (12 ± 3) nm, *Ar* = (55 ± 10) %. The best fit was obtained for *K*_D_ = 10, showing a high affinity of the GM_1_ probes for the domains. In contrast, the distribution of the CF-PEG-DSPE/Rh-PEG-DSPE D/A pair in the bilayer was not affected by the presence of nanodomains (*K*_D_(D,A) = 1), which did not allow for the detection of nanodomains by means of this D/A pair (see Table 1 and overlapping decays in the bottom left panel of Fig. 3).

It is worth noting that binary DOPC/Chol mixtures exhibit different behavior. We showed previously that lipid mixtures of DOPC/Chol (65/35) were homogeneous as determined by FRET^24^. Transient nanodomains were found for this binary mixture only close to the phase separation boundary by other methods^29, 30^ where miscibility of Chol with DOPC is low^28^ and Chol starts to phase-separate into anhydrous and monohydrate crystals^31^.

#### Nanodomains outside the L_d_/L_o_ phase coexistence region in DOPC/Chol/SM

Addition of 25 mol% of Chol to the DOPC/SM bilayers promoted the formation of nanodomains. Here nanodomains were detected at DOPC/Chol/SM (70–65/25/5–10) by g-GM_1_/r-GM_1_ D/A pair. The enhanced relative FRET efficiency as compared to the homogeneous DOPC/Chol/SM (75/25/0) bilayer and the time resolved fluorescence decays of the donor g-GM_1_ can be seen in Table 1 and Fig. 3, respectively. Determination of domain sizes by MC-FRET yielded an average *R*_D_ (9 ± 1) nm and *Ar* = (45 ± 5) %. Deep chi-squared minima were only reached when *K*_D_(D) and *K*_D_(A) were at least 20, demonstrating that the GM_1_ probes were highly localized in the nanodomains (see Fig. SI4). Moreover, the nanodomains were also detected by the CF-PEG-DSPE/Rh-PEG-DSPE DA pair at DOPC/Chol/SM (65/25/10) and (63/25/12) (Table 1 and Fig. 3). The affinity of the PEG-DSPE probes for the domains was lower (*K*_D_(D) and *K*_D_(A) ≈ 5), but sufficient to cause a change in the relative FRET efficiency and enable the determination of domain sizes at the higher SM amounts. The determined average *R*_D_ = (8 ± 1) nm and *Ar* = (55 ± 5) % are in good agreement with the parameters determined using the g-GM_1_/r-GM_1_ pair.

### Supportive evidence for nanodomain existence by z-scan FCS

**Figure 4:**
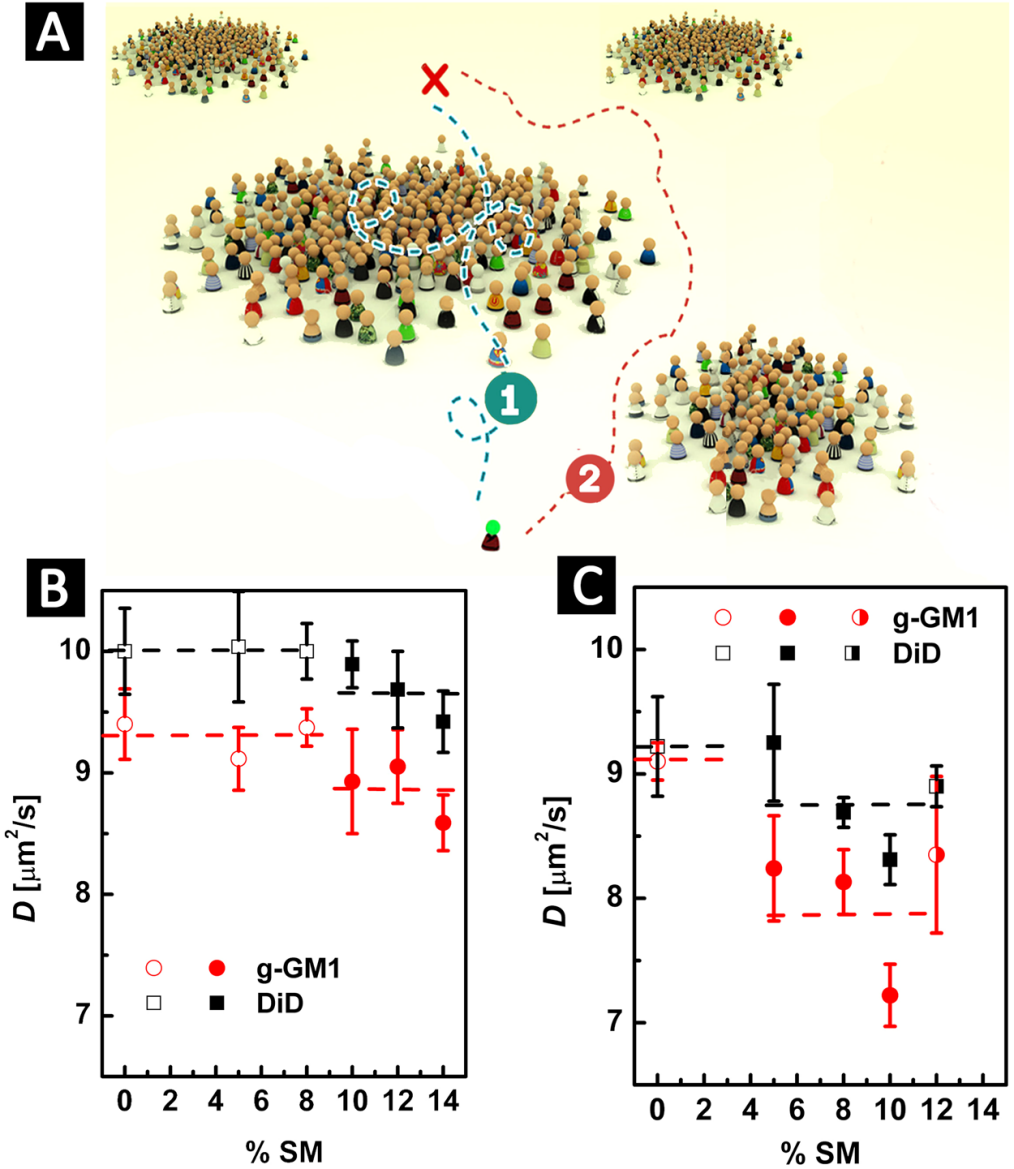
This figure demonstrates the impact of nanodomains on the diffusion of fluorescent probes. (**A**) Two scenarios are possible: the probes diffuse through the nanodomains (case 1); the probes avoid entering the nanodomains (case 2). At sufficiently high domain concentration, the probes diffuse significantly slower as compared to the case where the probes diffuse freely in a homogeneous bilayer^32^. Panels (B) and (C) show experimental results of two-colour z-scan FCS measurements performed on GUVs. The diffusion coefficients of g-GM_1_ and DiD are presented as a function of SM content in bilayers of DOPC/SM [(100 − x)/x] (panel B) or DOPC/Chol/SM [(75 − *x*)/25/*x*] (panel C). Empty vs. filled symbols mark bilayer mixtures where a homogeneous bilayer vs. a bilayer with nanodomains was detected by FRET. Microscopically phase-separated bilayers are marked by the half-filled symbol. Error bars are the standard deviation within the sample of results (measurements on 5 to 10 different GUVs) obtained for each composition.

Our FRET measurements indicate that the nanodomains occupy up to 55% of the entire bilayer area in binary DOPC/SM as well as ternary DOPC/Chol/SM lipid mixtures and exhibit an average radius of approximately 10 nm. According to our previous work focusing on MC simulations of molecular probe diffusion in a lipid bilayer^32^, the presence of stable (ca. >10 ms) nanodomains at such high domain concentration slows down the diffusion of fluorescently labeled lipids (=probes, Fig. 4). The extent to which the diffusion of the probes is slowed down depends in particular on their *K*_D_, the size of the nanodomains, the diffusion coefficient of the nanodomains themselves *D* (nanodomain), and the diffusion coefficient of the probes within those nanodomains *D* (probe). The strongest impact on probe diffusion occurs when the nanodomains are immobile and the probes have high affinity for them. When using classical FCS (where the focal waist is much larger than the nanodomains, about 300 nm vs. 10 nm) the presence of nanodomains is reflected in a slower diffusion behavior of the probe that can still be described by the free diffusion model (for example at *K*_D_(probe) = 25, domain radius = 50 nm and diffusion coefficient of nanodomains = 0.8 μm^2^/s probe diffusion is 5 times slower)^32^. However, considering the small size of the nanodomains described in this manuscript, it can be expected that they are mobile. In such case their impact on probe diffusion is less pronounced but still significant in most cases (for details see ref. 32). When probes avoid entering the nano-domains (*K*_D_ < 1, panel A of Fig. 4) their diffusion is slowed down as well and does not exhibit any deviations from free diffusion as seen by classical FCS. In general, their sensitivity to the presence of nanodomains is smaller compared to probes that partition mostly towards nanodomains.

Considering the high affinity of g-GM_1_ for nanodomains (Table 1), we used it as a probe to detect nanodomains by FCS. For comparison, we also used the DiD probe. It can be inferred from FRET experiments using g-GM_1_ and DiD (Fig. SI3), that DiD is homogeneously distributed between the nanodomains and the remaining bilayer. Therefore, the expected impact of nanodomains on its diffusion will be smaller.

In DOPC/SM lipid mixtures (panel B of Fig. 4) the dependence of the diffusion of g-GM_1_ on SM content in the lipid bilayer could be divided into two regimes: In the first regime, at DOPC/SM (100–92/0–8), the diffusion coefficients were constant within the error of the FCS measurement. In this regime, our FRET experiments showed a homogeneous bilayer. At DOPC/SM (90–85/10–15), where FRET detected nanodomains, the g-GM_1_ diffusion coefficient decreased. A similar trend but with slightly less distinct differences between the two regimes was obtained for DiD. According to panel B of Fig. 4, the diffusion of g-GM_1_ was on average about 5% slower in bilayers with nanodomains than in homogeneous bilayers, whereas the diffusion of DiD slowed down on average about 3%. In order to judge the significance of the decrease in the diffusion coefficient *D* a t-test was performed (see Table SI2 and SI3 in SI). *P*-values lower than 0.1 determine a significant difference between two sets of data. In case of g-GM_1_ the change in *D* in respect to the composition DOPC/SM (100/0), which contains no nanodomains, was significant for the compositions (90/10) and (85/15) and insignificant for (88/12). In case of DiD the drop was significant only for (85/15).

The impact of nanodomains on the diffusion of g-GM_1_ was much stronger in DOPC/Chol/SM mixtures (panel C of Fig. 4), where g-GM_1_ partitioned into the nanodomains more efficiently (see Table 1 for *K*_D_s), compared to the DOPC/SM bilayers. A significant drop in the diffusion of g-GM_1_ (see Table SI3 in SI) occurred already at (70/25/5), where nanodomains were detected by FRET. The diffusion slowed down further as more SM was accumulated into the nanodomains (note that the average domain radius and area coverage remain the same (Table 1) as SM content is increased). Based on the similarity of the hydrophobic molecular regions of SM and g-GM_1_, and the results of the MC simulation of probe diffusion in the presence of nanodomains^32^ this can be explained by more efficient entrapment (longer dwell-time) of g-GM_1_ in the SM-rich nanodomains. The abrupt increase in the diffusion coefficient at (63/25/12) occurred due to formation of microscopically phase-separated domains and concentration of Chol and SM into such domains. The bilayer was very close to the L_d_/L_o_ phase separation boundary at this composition. Here, there is an increased risk of measurements being unintentionally performed on phase-separated GUVs, where the diffusion in the L_d_ phase is fast^33^. This is also reflected in the large standard deviation error bar associated with this data point (panel C of Fig. 4). A similar pattern of diffusion coefficients was obtained for DiD. For comparison, the diffusion coefficient of g-GM_1_ decreased on average about 14% in bilayers containing nanodomains, whereas DiD diffusion was slowed down on average about 5%. Of note, the decrease in *D* of DiD in (67/25/8) and (65/25/10) bilayers was determined to be significant by the t-test (Table SI3).

### Nanodomain fluidity

According to the published phase diagrams^27, 28^, the investigated bilayers (Table 1) should be homogeneous and in a neat liquid-disordered state. However, we observed nanodomains under these conditions, thus, we questioned to which extent these nanodomains were fluid and disordered. We calculated the number of individual lipid molecules in the nanodomains assuming an equal distribution of DOPC between domains and remaining bilayer and an exclusive localization of Chol and SM within the nanodomains (see SI for details of this calculation). In DOPC/SM (90/10) bilayers the nanodomains contained on average approximately 94 SM and 390 DOPC molecules. This results in a SM to DOPC molar ratio within the nanodomains of 1:4. In DOPC/Chol/SM (65/25/10) bilayers, the nanodomains are estimated to contain 228 DOPC, 195 Chol and 78 SM molecules. This yields a 1:3 SM to DOPC molar ratio and a 1:2.5 SM to Chol molar ratio. In both DOPC/SM and DOPC/Chol/SM the number of DOPC molecules by far exceeds the number of SM molecules in the nanodomains. This makes the nanodomains fluid and disordered. Even in the case of the ternary mixture, where Chol molecules also contribute by a large fraction, the large amount of DOPC maintains the fluidity and disorder of the nanodomains. Moreover, since the DOPC/Chol/SM bilayer phase-separates at (60/25/15), these given numbers represent the approximate highest ratios of SM/DOPC and SM/Chol at which liquid disordered nanodomains could still be formed.

FRET experiments between two additional D/A pairs consisting of DOPE labeled at the headgroup by Atto-488 or Atto-633 (Atto-488-DOPE/Atto-633-DOPE), respectively, and between g-GM_1_ and the lipid tracer DiD further confirmed the liquid-disordered character of the nanodomains (Figs SI2 and SI3 in SI). These experiments are based on the assumption that by knowing the affinity of donors and acceptors for microscopically phase separated L_o_ domains, for which *K*_D_s can be determined easily, one can draw conclusions about the L_o_ character of nanodomains of similar composition by simple time-resolved FRET measurements (see SI). Here we are under the assumption that the lipid compositions, which are slightly different between the microscopic and nanoscopic domains, do not change *K*_D_s of probes. As shown in Table 2, Atto-488-DOPE, Atto-633-DOPE and DiD partition preferably into the L_d_ phase and are efficiently expelled from microscopic L_o_ domains in DOPC/Chol/SM (55/25/20) bilayers. This composition is close in the phase diagram (Fig. 2) to those where nanodomains are observed (e.g. DOPC/Chol/SM 65/25/10). Based on these results, one expects that these probes should be driven out of nanodomains with L_o_ character. This would change the relative FRET efficiency for both D/A pairs (see Table 2 for implications in FRET in the presence of L_d_ or L_o_ nanodomains). On the other hand, formation of L_d_ nanodomains would not change either the distribution of the probes or the relative FRET efficiency for both DA pairs. As Figs SI2 and SI3 show, the kinetics of fluorescent relaxation of the donors remained the same for all the cases. This indicates that the relative FRET efficiency for both D/A pairs remained constant in all investigated bilayer compositions, with and without nanodomains. This finding supports our hypothesis that the nanodomains have a L_d_ character.

**Figure 5:**
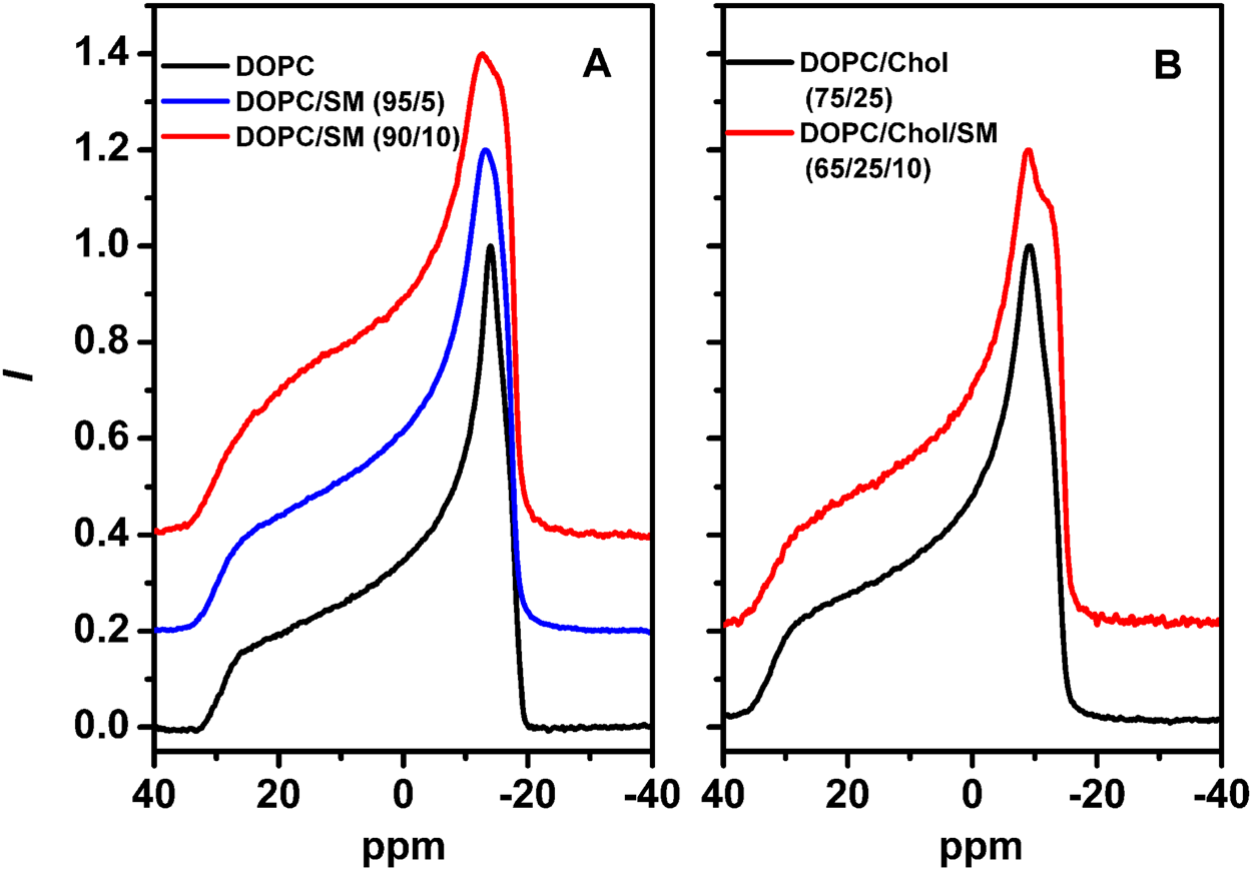
Static ^31^P NMR spectra of multilamellar vesicles composed of (left panel) DOPC (black), DOPC/SM 95/5 (red) and DOPC/SM 90/10 (blue) or (right panel) DOPC/Chol 75/25 (black) and DOPC/Chol/SM 65/25/10 (red) at 298 K.

To obtain further insight at a molecular level into the organization and dynamics of the lipid bilayers studied here, additional solid-state wide-line and high-resolution MAS ^31^P NMR experiments were performed^34, 35^. Wide-line NMR spectra obtained for pure DOPC bilayers (Fig. 5) exhibited a “powder-like” lineshape at 298 K, which is typical for a lamellar PC bilayer in its liquid-crystalline L_d_-phase^36, 37^. Under these conditions, the individual lipid molecules in the bilayer undergo fast rotational dynamics, which causes the typical shape and reduced width of the obtained NMR spectra. Analysis of the lineshapes revealed a chemical shift anisotropy, Δ*σ*, where Δ*σ* = *σ*_∥_ − *σ*_⊥_ is the width of NMR spectrum, of approximately 45.3 ppm, which is typical for this phase. Addition of 25 mol% of cholesterol to the DOPC bilayers generated a spectrum representative for a lamellar bilayer system at 298 K (Fig. 5B) with a hint of a second subspectrum, i.e. showing a homogeneous bilayer with perhaps a small fraction of a second, slightly more ordered subdomain. In contrast, the sample composed of DOPC/SM (90/10) resulted in significant changes in the corresponding NMR lineshape (Fig. 5A). The NMR spectrum is clearly composed of two sub-spectra, the main component with an intense 90° edge at −14.6 ppm and a second at −18.3 ppm. As SM is a minor component (10%) in the lipid bilayer, we attribute these two sub-spectra to DOPC in two different dynamic environments. Fitting the lineshape to two axially symmetric powder patterns reveals that the first sub-spectrum, characterized by a chemical shift anisotropy of 38.4 ppm, contributes approximately 47% of the total intensity. This component reflects a lipid environment with a slightly increased disorder in the headgroup region of the DOPC lipids. The second sub-spectrum with an intense 90° edge at 16 ppm, is characterized by a larger chemical shift anisotropy of approximately 49.0 ppm. This increase indicates a different membrane environment with the lipid headgroup regions (and presumably the whole lipid molecules) undergoing reduced dynamics (with less motional averaging of the chemical shift anisotropy) presumably due to the close proximity of stiff SM molecules. In summary, the DOPC/SM (90/10) membranes appear to consist of two different environments: 47% of the bilayer was presumably SM free, fluid and disordered whereas the rest of the bilayer was richer in SM with DOPC lipids in direct contact with SM molecules. Such estimation is in agreement with our MC-FRET results, according to which the SM rich domains occupied 37% or 55% (two global chi-squared minima) of the entire bilayer area (Table 1). Also in agreement with FRET data, 5 mol% SM was not able to induce nanodomains as seen in Fig. 5A, which would have been visible as a second NMR sub-spectrum characterized by a larger chemical shielding anisotropy.

For the ternary systems composed of DOPC/Chol/SM (70,65/25/5,10) lipid mixtures, static NMR spectra were clearly composed of two sub-spectra with different widths (Fig. 5B). Although multiple components (subspectra) are present in the system the spectral properties of each are inconsistent with the broader spectra that would be expected of lipids in their gel phase. The broader of these two components has an intense 90° edge at −14 ppm, whilst the remaining component has a smaller chemical shift anisotropy similar to that of DOPC/Chol bilayers in the absence of SM. Fitting the lineshape to two axially symmetric powder patterns indicates that the SM rich contributes approximately 44% of the entire spectral intensity in good agreement with MC-FRET results.

**Figure 6:**
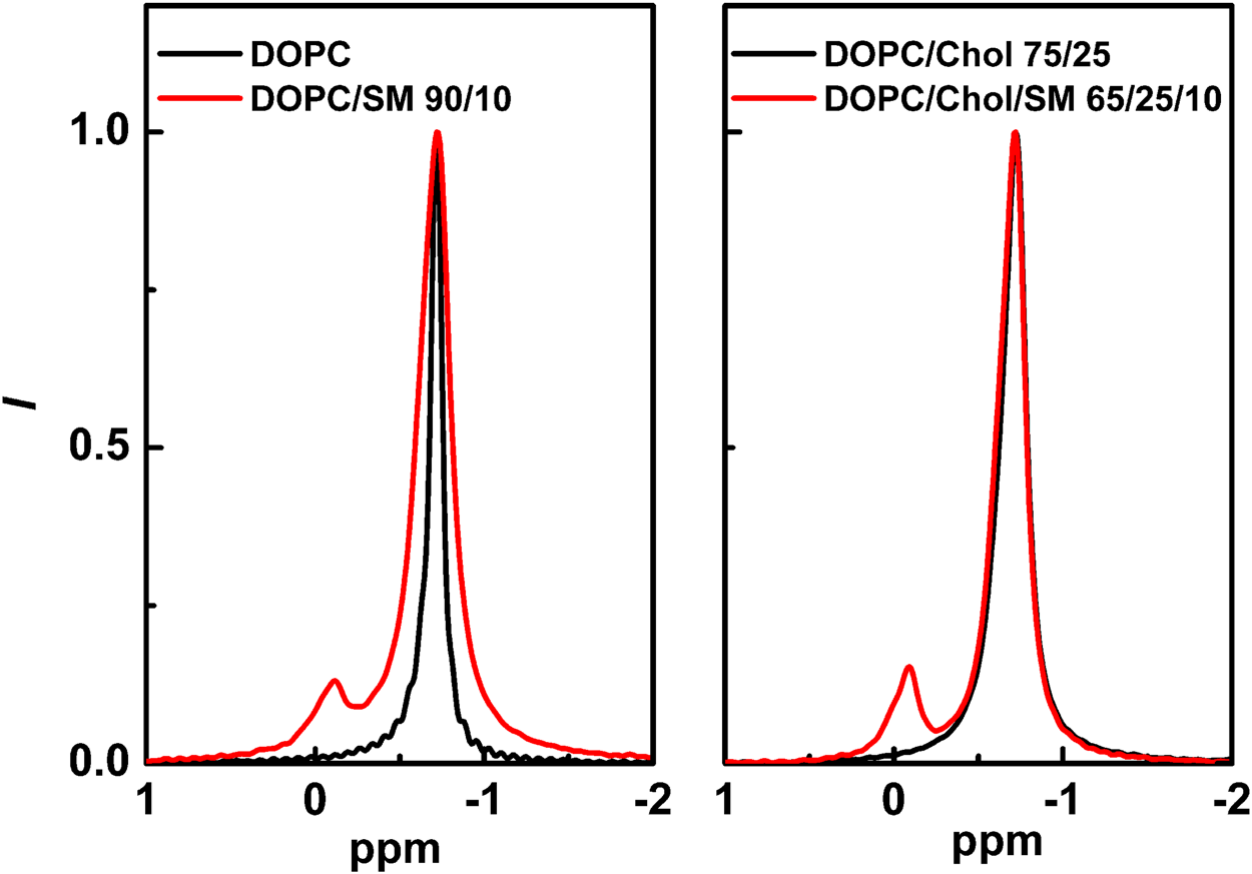
Change in the MAS ^31^P NMR spectra after addition of 10% of SM into DOPC (left panel) or DOPC/Chol (75/25) (right panel) lipid mixtures.

Our MAS ^31^P NMR results further confirm the L_d_ character of the nanodomains found in the studied bilayers. As seen in Fig. 6, the isotropic NMR signal occurred at −0.71 ppm, which is the isotropic chemical shift value expected for DOPC bilayers. Upon addition of SM, or Chol and SM, the variations in this value were minor (upfield to −0.74 ppm) but the NMR linewidth increased dramatically from 15 Hz to 45 Hz. Despite the increase in the linewidth, the resonances remained relatively narrow, supporting the disordered character of the lipid headgroups within the domains. The lipids are undergoing fast dynamics and the isotropic lineshapes of the MAS spectra are influenced by exchange processes. Such behaviour is also typical for L_d_ phase. The isotropic linewidths in the MAS ^31^P NMR spectra of lipids are largely dominated by the spin-spin/transverse relaxation times which are sensitive to motion on the ms to µs timescale^38^. The similarity of the linewidths obtained for DOPC in the presence of SM, Chol or both SM and Chol indicates that both species are likely to interact with DOPC headgroups, slowing down the motions on the ms to µs timescale and resulting in reduction of the *T*_2_ and an increase in the corresponding linewidth. The absence of more significant changes in the ^31^P powder lineshape reflects that the exchange processes occurring do so from populations of lipids exhibiting similar isotropic and anisotropic chemical shielding suggesting that both populations exhibit similar dynamic properties.

The fact that the nanodomains were detected both by NMR and FCS helps to restrict the range of possible nanodomain lifetimes. The readouts of both techniques are influenced by processes that occur on the micro- to millisecond time scale. Therefore, the lifetimes of nanodomains should be roughly in this range. The broadening that is present in the SM/DOPC MAS ^31^P NMR spectra (Fig. 6, red colour) together with the underlying broad subspectrum (ranging from 0.3 to −1.5 ppm) indicates that the DOPC is in exchange between different environments on the NMR timescale (ms/µs) in a manner analogous to that reported by e.g. by Bonev *et al*.^39^. This observation is consistent with SM lipids forming a dynamic complex with multiple DOPC molecules over the timescale of the NMR experiment (ms/µs). Interestingly, g-GM_1_, r-GM_1_, CF-DSPE-PEG and Rh-PEG-DSPE probes seem to exhibit increased affinity for this dynamic complex (see Table 1 for *K*_D_s). Preferable localization of these probes in the nanodomains originates presumably from structural similarity of hydrophobic regions of these probes with SM and allows for determination of nanodomain sizes by MC-FRET.

Of note, transient and dynamic heterogeneities of much shorter lifetime, about 100 ns, and size of 10 nm were revealed by neutron scattering^29, 40^ and MARTINI simulations^41^. These were reported to exist in DPPC/Chol and DMPC/Chol bilayers close to the phase separation boundary where Chol crystals start to form. Moreover, longer lived fluctuations in composition of about 0.8 ms were observed in ternary mixtures of high and low melting temperature lipids near miscibility critical points^42^. In this work, on the contrary to the above mentioned cases, the nanodomains are found further away from the phase separation boundaries (Fig. 2).

Although the mechanism of how these nanodomains are formed is not yet well understood, we expect that the process is facilitated by the following factors. First, geometrical factors result in different packing preferences of DOPC and SM. Consequently, SM tends to be surrounded by other SM rather than DOPC molecules. Moreover, it has been documented by a variety of experimental approaches that SM and Chol preferably interact with each other (for a review see ref. 43). It is also known that Chol promotes segregation of different PC components at low Chol contents whereas it suppresses the segregation at higher concentrations (above 50 mol%)^44^. All these interactions seem to be re-enforced by hydrogen bonding between –NH group of SM and hydroxyl group of Chol and between SM and DOPC^43, 45–47^. In addition, temporal thermal fluctuations and fluctuations in concentration may perhaps be involved in the formation of nanodomains^41^. Groupings of three to five molecules have been found even in ideal binary mixtures with only nearest neighbor interactions^48^. Thus, hypothetically, it is possible that these temporal fluctuations function as seeds for the liquid disordered nanodomains in a similar way as nanodomains work as formation platforms for microscopic L_o_ phase domains.

### Implications for the raft theory

So far, scientists have frequently used L_o_ microdomains as a model system for rafts despite insufficient experimental evidence for the existence of such domains in cells. The size and physical properties of such microdomains are extreme in the context of the plasma membrane, but they are still used as a model system for putative nano-scale rafts in cellular membranes. In respect to the complex composition of a cellular plasma membrane where sharp and well-defined phase transitions can hardly be expected, differences between various local environments are presumably more subtle than the differences between the L_d_ and L_o_ phases encountered in model systems. To bridge the gap between the extremes of L_d_ or L_o_ phase bilayers in artificial model systems and the plasma membrane of living cells scientists started using giant plasma membrane vesicles (GPMVs) as an intermediate model system that more or less preserve biological complexity of native plasma membranes^5, 49^. Similarly to the synthetic model membranes of GUVs, GPMVs may phase separate into two distinct microscopic phases. However, the differences between the L_d_ and L_o_ phase are significantly smaller to those observed in GUVs, presumably better corresponding to what is encountered in plasma membranes of living cells. A disadvantage might be a worse control of the GPMVs’ composition^6^ and phase behaviour which is dependent on the detergent used^49^.

The L_d_ nanoscale domains that have been found and characterized in this work have in analogy to the L_o_ microdomains encountered on GUVs a very simplified composition. However, in terms of domain sizes and their physical properties the nanodomains seem to represent a good model system for cellular rafts. Moreover, the plasma membrane of a living cell is permanently changing. Thus, rafts can be expected to form and disappear or change their properties during their lifetime. In this context, the transient nature of the L_d_ nanodomains may also correspond better to the properties of cellular rafts.

### Summary

In this work, we discovered and characterized nanodomains in binary DOPC/SM and ternary DOPC/Chol/SM bilayers of compositions that should result in homogeneous bilayers according to published phase diagrams. The results of our MC-FRET, solid-state NMR and z-scan FCS experiments are summarized in the phase diagram of Fig. 2 and in Table 3. Briefly, all three methods indicate that binary mixtures DOPC/SM (100–92/0–8) are homogeneous and that DOPC/SM (90–85/10–15) exhibit nanodomains. In the ternary lipid mixtures containing Chol, nanodomains were revealed at DOPC/Chol/SM (70–65/25/5–10).

**Table 3:** Nanodomains as detected by FRET and two different D/A pairs, solid-state NMR and z-scan FCS.

Composition of the lipid bilayer			Nanodomains detected YES/NO					Area covered by the nanodomains [%]	
DOPC [%]	SM [%]	Chol [%]	FRET		NMR	FCS		FRET	NMR
			g-GM_1_/r-GM_1_	CF-PEG- DSPE/Rh-PEG-DSPE		g-GM_1_	DiD		
100, 95, 92	0, 5, 8	0	NO	NO	NO	NO	NO	no nanodomains	
90, 88, 85	10, 12, 15	0	YES	NO (*K*_D_ ≈ 1)^a^	YES	YES	NO/YES^b^	37, 55	53
75	0	25	NO	NO	NO	NO	NO	no nanodomains	
70, 67	5, 8	25	YES	NO (*K*_D_ ≈ 1)^a^	—	YES	NO/YES^c^	45	—^d^
60	10	25	YES	YES	YES	YES	YES	45–55^e^	44
63	12	25	NO (*K*_D_ ≈ 1)	YES	—^d^	YES	NO	45	—^d^

^a^This D-A pair is equally distributed between the nanodomains and the remaining bilayer and thus the existence of the nanodomains does not have an effect on the FRET phenomenon; ^b^the decrease in D, which indicates the presence of nanodomains, was only significant (p < 0.1) for the bilayers composed of 15% SM; ^c^only for 8% SM; ^d^not determined; ^e^45% with GM_1_ pair, 55% with DSPE pair.

The nanodomains of approximately 10 nm can be estimated to consist of roughly 400 to 500 molecules, are enriched in SM but still contain a high amount of DOPC molecules, which is sufficient to maintain the nanodomains fluid and disordered. Despite their L_d_ character the nanodomains exhibit subtle differences on average environment and dynamics as compared to the surrounding. The nanodomains appear long-lived with a lifetime in the range of microseconds to several milliseconds. In terms of their size, fluidity, order and lifetime these nanodomains may represent a relevant model system for cellular membranes and perhaps be more closely related to heterogeneities, e.g. nanocompartments, observed in cellular plasma membranes.

## Methods

### GUV preparation

GUVs were prepared by the electroformation method as described previously by Angelova *et al*.^50^. All lipid mixtures were made from stock solutions in chloroform. The lipid mixture (100 nmol in approximately 200 μL of chloroform) containing the additional labelled lipids was spread onto two hollowed titanium plates. These were placed on a heating plate at approximately 47 °C to facilitate solvent evaporation. The plates were subsequently put under vacuum for at least 1 h to evaporate remaining solvent traces. The lipid-coated plates were assembled using one layer of Parafilm as an insulating material. The electroswelling chamber was filled with 1 ml of preheated sucrose solution (with the osmolarity of 103 mOsm/kg) and sealed with Parafilm. An alternating electrical field of 10 Hz rising from 0.02 V to 1.1 V (peak-to-peak voltage) during the first 45 min was applied and kept at 1.1 V and 47 °C for additional 1.5 h. This sequence was followed by a so-called detaching phase at 4 Hz and 1.3 V for 30 min. Finally, the GUVs were added to a microscope chamber containing glucose buffer (~80 mM glucose, 10 mM HEPES and 10 mM NaCl, pH 7.2) at the osmolarity of 103 mOsm/kg. All lipid mixtures contained 2 mol% of biotinyl-PE to immobilize the GUVs on the bottom of a chamber coated with BSA-biotin/streptavidin.

For the FCS experiments, the probe-to-lipid ratio was 1:100000 whereas for the FRET experiments, the donor (acceptor)-to-lipid ratio was 1:1000 (1:200) in case of g-GM_1_/DiD pair and 1:200 (1:200) in case of g-GM_1_/r-GM_1_ pair, respectively.

### Sample preparation for NMR experiments

The lipid mixtures were prepared by dissolving the appropriate lipids in a 2/1 vol/vol HCCl_3_/MeOH solution, followed by evaporation, resuspending in water and freeze-drying, as described previously^51^. To produce multilamellar vesicles, appropriate amounts (around 20 mg) of dry lipid powder was then rehydrated using in the same buffer as used above (except D_2_O was used here instead) at a one-to-one weight ratio, followed by several freeze-thaw cycles and vortexing. Finally the membrane suspensions were pelleted into 4 mm MAS NMR rotors (Bruker, Germany) and measured immediately or kept at −20 °C prior NMR experiments.

### FCS and FLIM-FRET measurements

Both types of measurements were performed on a home-built confocal microscope consisting of an inverted confocal microscope body IX71 (Olympus, Hamburg, Germany) and pulsed diode lasers (LDH-P-C-470, 470 nm, and LDH-D-C-635, 635 nm PicoQuant, Berlin, Germany) operated at 10 MHz repetition rate. The lasers were pulsing alternatively to avoid artifacts caused by signal bleed-through. The laser light was coupled to a polarization maintaining single mode optical fiber and re-collimated at the output with an air space objective (UPLSAPO 4X, Olympus). The light was up-reflected to a water immersion objective (UPLSAPO 60x, Olympus) with a 470/635 dichroic mirror. The signal was split between two single photon avalanche diodes using 515/50 and 697/58 band pass filters (Chroma Rockingham, VT) for green and red channel, respectively.

z-scan measurements were conducted on the top of selected GUVs. First, a membrane was placed to the waist of a laser, moved 1.5 µm below the waist afterwards and finally, scanned vertically in 20 steps (150 nm spaced). A 60 second long measurement was performed at each step. The laser intensity at the back aperture of the objective was around 6 µW for each laser line. To obtain the average diffusion coefficients presented in Fig.4 z-scan FCS measurements on 5–10 different GUVs were performed. Further details of the data analysis are given elsewhere^19^.

FLIM-FRET measurements were done by acquiring an image (512 × 512 pixels, 0.6 ms/pixel) of a GUV at its cross-section. The experimental fluorescence decay of the donor that was taken for further analysis was obtained by summing up the measured fluorescence decays from at least five different GUVs. However, variability between fluorescence decays obtained from individual GUVs was negligible (see Fig. SI6). Laser intensity of 1 µW for the 470 nm laser was chosen low enough to avoid pile-up effect for the FLIM-FRET measurements. The experiments were performed at 25 °C.

### NMR experiments

All ^31^P NMR experiments were acquired using a 500 MHz Avance III spectrometer (Bruker, Switzerland). Static wideline NMR spectra of multilamellar vesicles were acquired at 298 K using a Hahn echo pulse sequence with a single π/2 pulse of 7.8 µs pulse length, an inter-pulse delay of 50 µs and a recycle rate of 4 s. During acquisition, TPPM proton decoupling^52^ was used (40 W) and ca. 10000 scans were accumulated. For high-resolution MAS NMR spectra, the samples were spun at 5 kHz and a single pulse excitation followed by proton decoupling (parameters as for static NMR experiments) was used. Between 200 to 600 scans were accumulated.

NMR data was processed in matNMR^53^, with all spectra zero-filled to 4096 pts and 30 Hz line-broadening added prior to Fourier transform. Powder lineshapes were analyzed by fitting to one or two axially symmetric powder patterns, using the fitting routines within matNMR.

### Analysis of FLIM-FRET data

Förster resonance energy transfer (FRET) was analyzed from fluorescence lifetime images (FLIM). Each pixel contains information on the arrival times of individual photons. These times are used to construct the fluorescence decay, whose shape can be modified by FRET. Analysis of the decay by an appropriate mathematical model yields further information. In this work, the so-called Baumann-Fayer (BF) model was used (see SI) to (i) determine the experimental surface concentration of the acceptors, which was required as one of the input parameters for the MC simulations; (ii) obtain information about how donors and acceptors were distributed in the lipid bilayer. Relative FRET efficiency *E*_rel_ used in the manuscript is defined as the ratio between FRET efficiency^54^ for a heterogeneous bilayer *E*_hetero_ (with nanodomains) and the FRET efficiency for a homogeneous bilayer *E*_homo_ (without nanodomains). Homogeneous bilayers were selected to contain 0% of SM and the same amount of Chol as the heterogeneous bilayers.

*The determination of nanodomain sizes* was performed by analyzing the experimental fluorescence decay with Monte Carlo simulations. Two sets of GUVs are always prepared: the first set contains GUVs with homogeneous bilayers and is used to calculate the number of acceptors in the GUVs by the Baumann-Fayer model (see SI). The number of acceptors is assumed to be the same in the other set of GUVs, where nanodomains with unknown dimensions might exist. This is done in order to reduce the number of optimized parameters. The entire fitting procedure was described in detail elsewhere^24^ and is shortly summarized in what follows. A defined number of donors, acceptors and circular domains with a given radius *R*_D_ was generated in the lipid bilayer. Whereas the number of donors was kept at a sufficiently high value for statistical reasons, the number of acceptors had to be determined by the BF model (SI) to correspond to the actual experimental conditions. First, the donors and acceptors were distributed according to the distribution constants defined as *K*_D_(D) = [D_inside_]/[D_outside_], *K*_D_(A) = [A_inside_]/[A_outside_]. In the next step, a donor was randomly excited and the time at which an energy transfer event took place calculated. This process was random and modulated by the overall energy transfer rate *Ω*_*i*_ according to Δ*t*_*i*_ = −*lnγ*/*Ω*_*i*_ where *γ* is a randomly generated number between 0–1. The outcome of each simulation step was the time interval Δ*t*_*i*_ between the excitation and the energy transfer event. To achieve good statistics, each generated configuration was used 100 times before a new configuration was generated. The total number of all excitation events was 3 × 10^5^. By constructing a histogram of Δ*t*_*i*_ intervals the total survival probability function *G(t)* was obtained and the simulated decay of donors quenched by the acceptors calculated. The simulated decay was fitted to the experimental one by varying the input simulation parameters, i.e. the domain radius *R*_D_, the area fraction the domains occupied *Ar* and *K*_D_(D,A). The global minimum was found by scanning the chi-squared space of physically acceptable parameters *R*_D_, *Ar*, and *K*_D_(D,A). Because of structural similarity between donors and acceptors and a weak dependence of *R*_D_ and *Ar* on the actual values of *K*_D_, *K*_D_(D) was kept identical to *K*_D_(A).

*Analysis of z-scan FCS data* has been described many times before^19, 55^ and is briefly summarized in SI.

## Electronic supplementary material

Supplementary Info
