# Supplementary material for: Lipid Driven Nanodomains in Giant Lipid Vesicles are Fluid and Disordered
Source: Sci Rep. 2017 Jul 14;7:5460. doi: 10.1038/s41598-017-05539-y (PMC5511215; doi:10.1038/s41598-017-05539-y)
Supplement: Supplementary file 1 — Supplementary Info [file 41598_2017_5539_MOESM1_ESM.pdf]

Lipid Driven Nanodomains in Giant Lipid Vesicles Are Fluid and Disordered: Supporting Information

Alena Koukalová, Mariana Amaro, Gokcan Aydogan, Gerhard Gröbner, Philip T.F. Williamson, Ilya Mikhalyov, Martin Hof and Radek Šachl

Baumann-Fayer model[1] describes a situation where donors and acceptors are homogeneously distributed in two parallel planes of a bilayer. In such a case, FRET occurs within one bilayer leaflet (*intra*-FRET) as well as between two parallel planes (*inter*-FRET). By assuming dynamic limit conditions the survival probability function for *intra*-FRET G_{intra} can be expressed as

$$ln\, G_{intra}(t) = -C_2 \Gamma\left(\frac{2}{3}\right)\left(\frac{t}{\tau}\right)^{1/3}, \qquad (1)$$

where C_2 is the reduced surface concentration of the acceptors, which represents the average number of acceptors surrounding a donor within an area of πR_0^2, Γ is the gamma function and τ the average lifetime of the donors. The survival probability for *inter*-FRET, G_{inter}, depends on C_2 but also the bilayer thickness d and is expressed as

$$ln\, G_{inter}(t) = -\frac{C_2}{3}\left(\frac{d}{R_0}\right)^2\left(\frac{2\mu}{3}\right)^{1/3}\int_0^{2/3\mu}(1-e^{-s})s^{-4/3}ds, \qquad (2)$$

where θ_r is the angle between the bilayer normal and the vector connecting the locations of the donor and acceptor dipoles, $\mu = 3t\left(\frac{R_0}{d}\right)^6\frac{1}{2\tau}$ and $s = 2\mu\cos^6\frac{\theta_r}{3}$. Both *inter*- and *intra*-FRET may occur simultaneously in a lipid bilayer. Therefore, the total survival probability of the donors is

given by the joint probability $G(t) = G_{\text{intra}}(t)G_{\text{inter}}(t)$ and the fluorescence intensity $F(t)$ of the donors in the presence of the acceptors described as

$$F(t) = G(t)\sum_i \alpha_i \, exp\left(-\frac{t}{\tau_i}\right), \qquad (3)$$

where $\sum_i \alpha_i \, exp(-t/\tau_i)$ represents the donor decay in the absence of FRET.

Table SI1: Comparison of the acceptor to lipid ratios determined by the Baumann-Fayer model with the expected acceptor to lipid ratios gives information about clustering of fluorescent probes.

DOPC	SM	Chol	Fluorescent probe	Acceptor to lipid ratio obtained by fitting	Expected acceptor to lipid ratio
100, 95, 92	0, 5, 8	0	r-GM_1	1:279*	1:200
75	0	25	r-GM_1	1:196	1:200

*The following lipid compositions provided identical time-resolved fluorescence decays. Therefore, one value is presented for more than one lipid composition.

FRET excludes intrinsic self-aggregation of GM_1 probes at the concentrations used. One way to exclude self-aggregation of GM_1 probes is to use the Baumann-Fayer model (see above). This model provides a parameter called reduced surface concentration of acceptors (C_2), which gives the number of the acceptors in a circle determined by R_0 and can be recalculated to yield the acceptor to lipid ratio. This value is a priori known by mixing lipids and probes with each other and can be compared with the value obtained by fitting. According to [2] aggregation leads to significantly (2.5 to 5 times) larger C_2 values obtained by fitting as compared with the expected values. In our experiments GUVs were prepared at the acceptor to lipid ratio 1:200. Self-aggregation can be excluded because values close to this number were obtained by fitting.

Results are summarized in Table SI1. Of note, this approach can be used only for probes distributed in homogeneous bilayers where Baumann-Fayer model can be applied.

In heterogeneous bilayers a qualitative approach which is based on FRET can be used instead. Here the concentration of donors is varied while keeping a constant concentration of acceptors. If no aggregation occurs overlapping time-resolved fluorescence decays should be obtained. According to Fig. SI1 no aggregation of GM_1 probes in DOPC/Chol/SM (65/25/10) bilayers that contain nanodomains occurs because all decays overlap perfectly.

In conclusion, these experiments indicate that GM_1 probes do not intrinsically self-aggregate at the lipid bilayers and concentrations we have used.

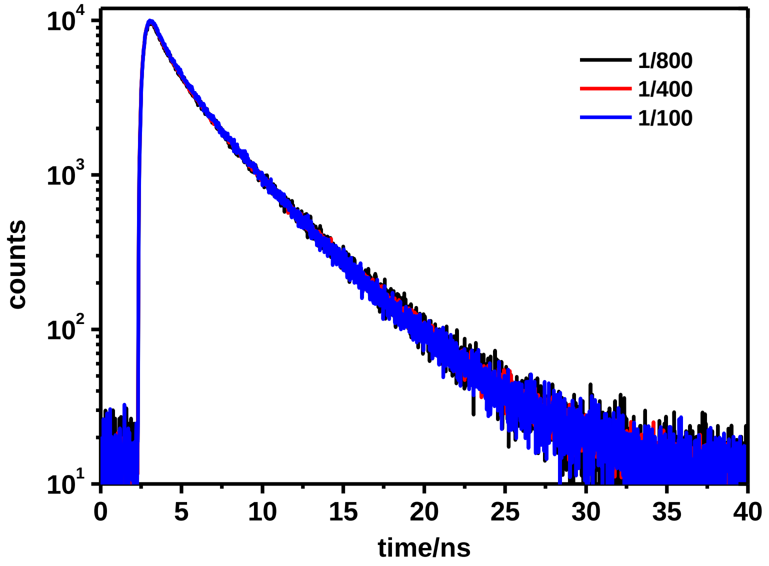

Fig. SI1: Time-resolved fluorescence decays of g-GM_1 in the presence of r-GM_1 acceptors in DOPC/Chol/SM (65/25/10) bilayers. The g-GM_1 to lipid ratio was varied from 1:100 to 1:800.

Determination of the distribution constant K_D between the L_o microdomains and the remaining bilayer. The probe preferences for L_o vs. L_d were determined on microscopically phase

separated giant unilamellar vesicles (GUVs) by fluorescence intensity measurements[3]. The fluorescence intensity was extracted from individual pixels that either belonged to L_o or L_d phase and averaged out to obtain the average fluorescence intensity for L_d, F_{Ld}, or L_o phase, F_{Lo}, respectively. Assuming that F is proportional to the dye concentration in the membrane, the following equation can be used for the calculation of K_D (for K_D definition see the manuscript)

$$K_D = \frac{F_{Lo}}{F_{Ld}+F_{Lo}}. \qquad (4)$$

According to[3], the correction for different brightness in L_d vs. L_o plays a minor role and was consequently not performed in this work, where a rough K_D determination was sufficient.

Nanodomain fluidity and order influences distribution of fluorescent probes. The affinity of fluorescent probes for the domains and the remaining bilayer usually differs. If the probe preferences (given by the partition coefficient K_D) are known *a priori* and under the assumption that the lipid composition does not influence K_Ds at least in a narrow range of lipid concentrations, the fluidity and order of the nanodomains can be characterized. For this purpose, we used two different donor-acceptor (D-A) pairs in this work:

1) Atto-488-DOPE/Atto-633-DOPE: According to Table 2 in the manuscript, both donors and acceptors have very low affinity for L_o microdomains. Formation of L_o nanodomains should therefore be manifested by an increased FRET efficiency because Atto-488-DOPE donors would come into more frequent contact with Atto-633-DOPE acceptors as the donors and acceptors would be more concentrated in the remaining L_d phase. However, the existence of nanodomains did not change the FRET efficiency (Fig. SI2), which implies that the distribution of donors and acceptors had to remain homogeneous. Not surprisingly, it followed from the MC-FRET analysis

that K_D(D, A) = 1, confirming the homogeneous distribution of the probes and the disordered character of the nanodomains.

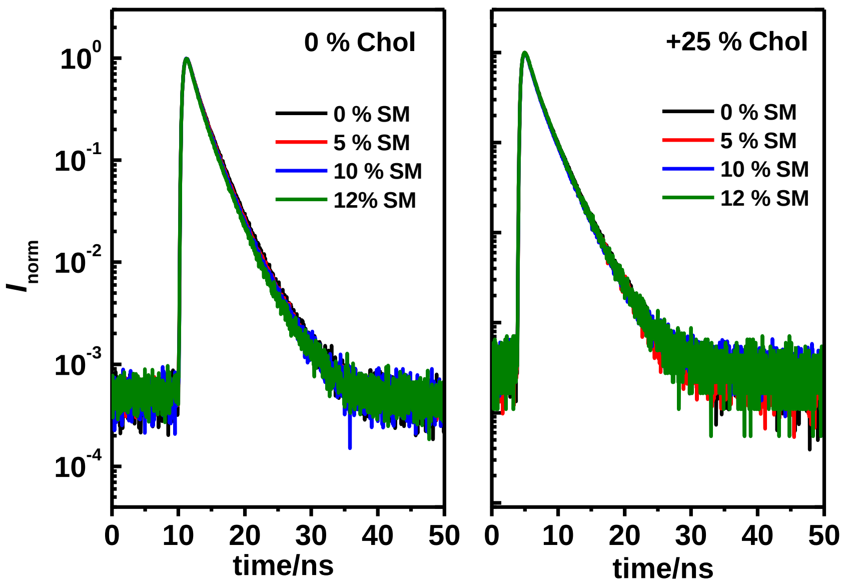

Figure SI2. Time-resolved fluorescence decays for Atto-488-DOPE in the presence of Atto-633-DOPE in DOPC/SM (100-*x*/*x*) (left panel) or DOPC/Chol/SM (75-*x*/25/*x*) (right panel) lipid mixtures with variable amounts of SM.

2) g-GM_1/DiD: Here, donors are accumulated in L_o regions of the bilayer whereas acceptors are efficiently excluded from them (Table 2 in the manuscript). Therefore, existence of L_o nanodomains should significantly decrease FRET efficiency, as the donors are driven apart from the acceptors. No such behavior was found in the time resolved fluorescence decays (Fig. SI3) as SM content in the bilayer was increased; thus further supporting the disordered character of the nanodomains.

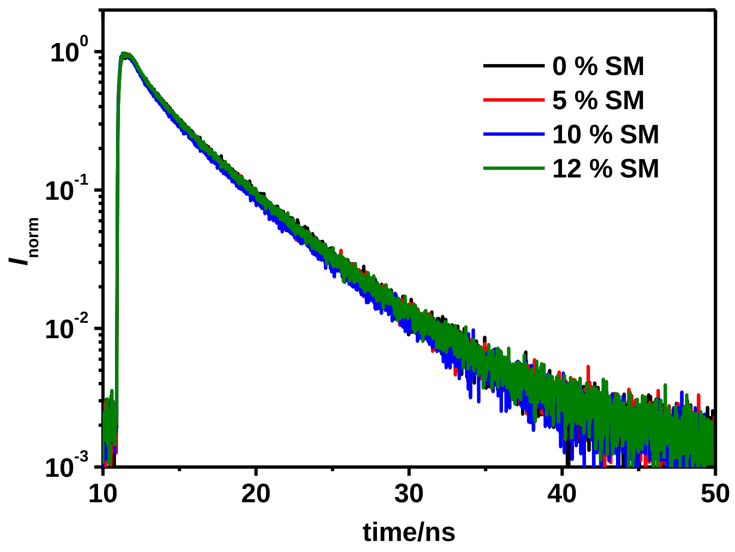

Figure SI3. Time-resolved fluorescence decays for g-GM_1 in the presence of DiD acceptors. The GUVs consisted of DOPC/Chol (75/25) (no nanodomains, black) or DOPC/Chol/SM (70-63/25/5-12) (containing nanodomains, red, blue, olive), respectively.

FRET as an indicator of bilayer condensation/expansion. It is important to realize that condensation of the lipid bilayer as well as accumulation of the probes in the nanodomains brings donors and acceptors closer to each other. As seen by FRET, these two phenomena may interfere with each other. For this reason, condensation of the lipid bilayer has to be considered when drawing conclusions about the nanodomain sizes and concentrations. In this work, no condensation of the bilayer occurred when SM content in the bilayer increased and the nanodomains formed. Therefore, no special corrections were necessary. This is documented by the constant FRET efficiency between Atto-488-DOPE and Atto-633-DOPE and between g-GM_1 and DiD as shown in Figs. SI2 and SI3.

FRET as a tool to measure size and concentration of the nanodomains. As described in detail in the manuscript as well as in[4], the MC-FRET approach is based on fitting time-resolved fluorescence decays with simulated decays whose shape depends, among other input simulation parameters, on the size and concentration of the nanodomains and K_Ds. Representative chi-squared maps are shown in Figs. SI4 and SI5. The maps in Fig. SI4 were made for such K_D for which the best fit to the experimental data was obtained. Minima in the chi-squared parameter were found in DOPC/Chol/SM (65/25/10) bilayer at the nanodomain radius R_D = 9 nm and the fractional domain area Ar = 45 % when using g-GM_1/r-GM_1 or at R_D = 8 nm and Ar = 55 % when using CF-PEG-DSPE/Rh-PEG-DSPE donor-acceptor pair. The maps of Fig. SI6 show the chi-squared values for four different K_Ds, demonstrating a fact that the position and depth of the global chi-squared minimum for the GM_1 probes in DOPC/Chol/SM (65/25/10) bilayers is practically constant for K_D $\geq$ 20.

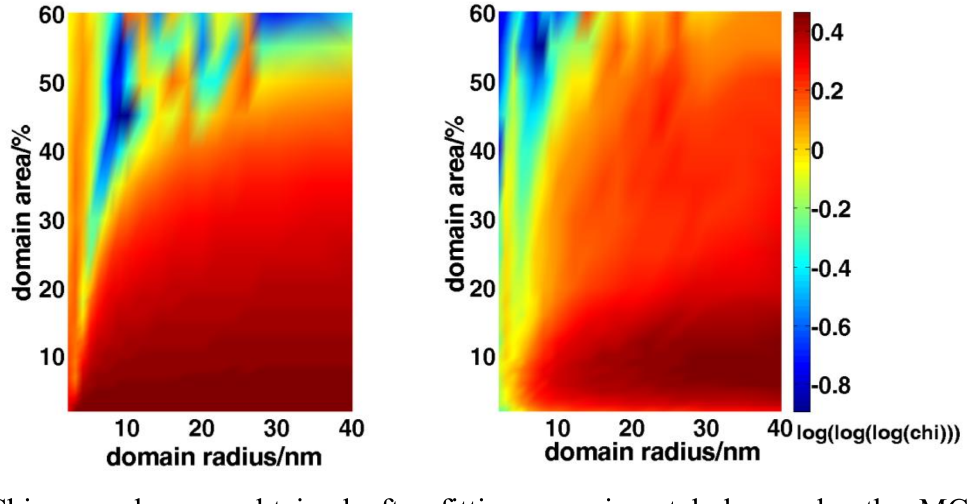

Figure SI4. Chi-squared maps obtained after fitting experimental decays by the MC-FRET approach. The lipid mixture consisted of DOPC/Chol/SM (65/25/10). The domain sizes and concentration were determined by either g-GM_1/r-GM_1 (left panel) or CF-PEG-DSPE/Rh-PEG-DSPE (right panel) donor-acceptor pair. The chi-squared maps are shown for K_D(g-GM_1, r-GM_1)

= 1000 and K_D(CF-PEG-DSPE, Rh-PEG-DSPE) = 10, i.e. for such K_D values for which the best fit to the experimental data was obtained. To improve visibility of the chi-squared minima, *chi*, log(log(log(*chi*))) is displayed in the figure.

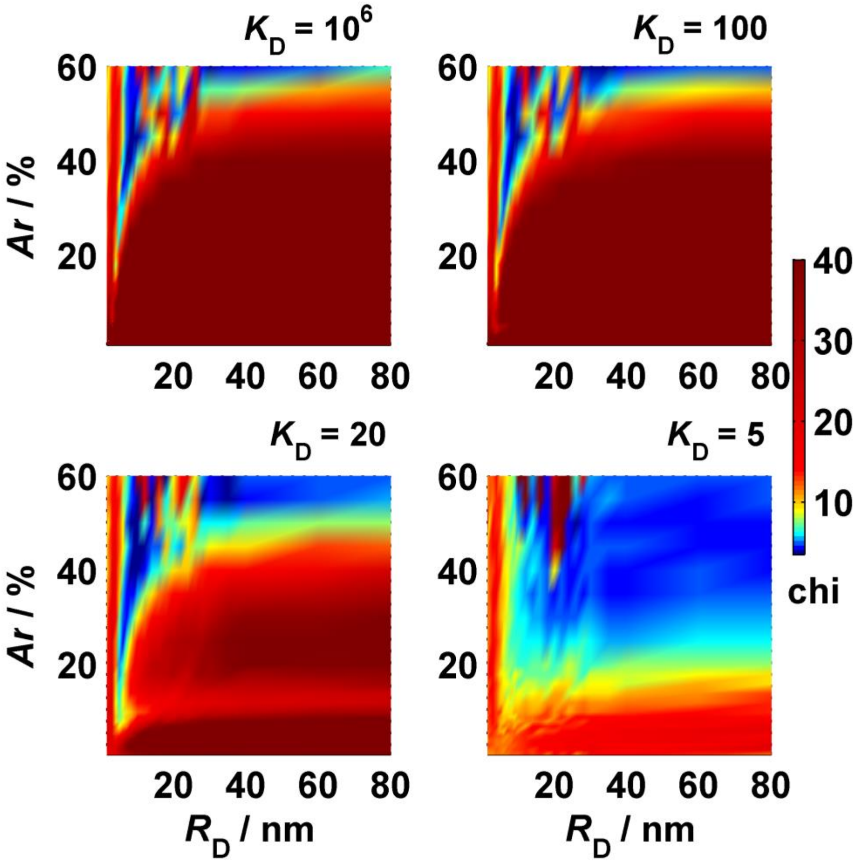

Fig. SI5: chi-squared minima (chi) for a set of different distribution coefficients K_D of g-, r-GM_1 probes. Specifically, the global minimum in chi-squared for K_D = 20 is only about 0.5 % lower than the minimum for K_D = 1000. The lipid mixture consisted of DOPC/Chol/SM (65/25/10).

Variability of nanodomain size and the fractional area occupied by the nanodomains between individual GUVs. Interestingly, variability in the nanodomain size and concentration between individual GUVs was extremely low. This can be documented by overlapping time-resolved fluorescence decays obtained from different GUVs of the same sample (see Fig. SI6). These decays were used to calculate the size and fractional area of the nanodomains in lipid

bilayers

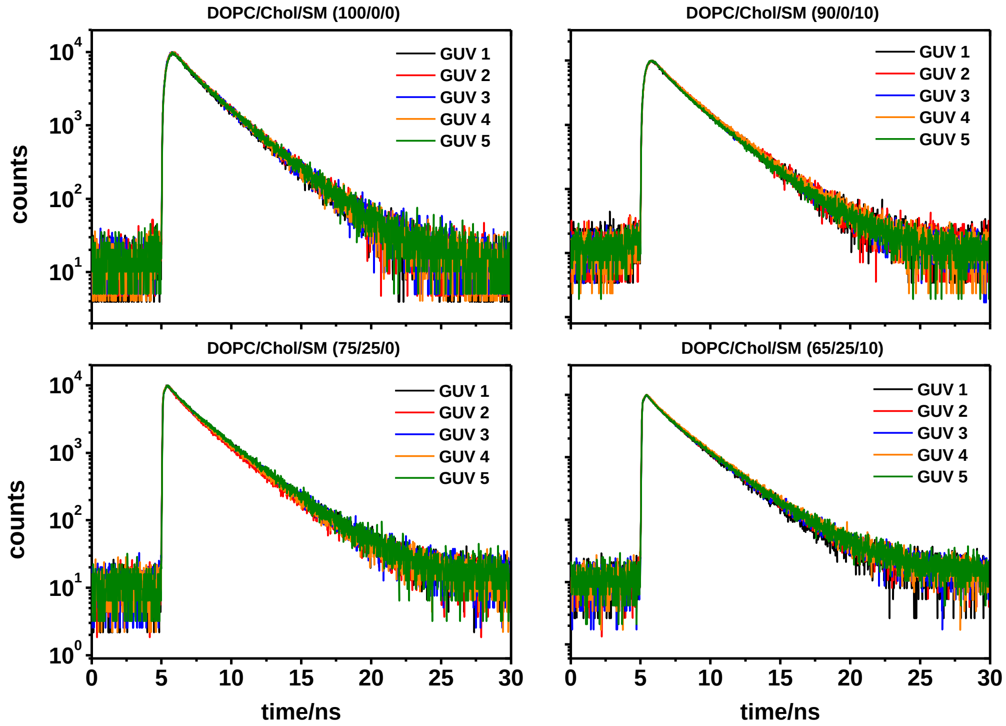

Figure SI6: Time-resolved fluorescence decays of CF-DSPE-PEG in the presence of Rh-DSPE-PEG acceptors obtained from individual GUVs for four different bilayer compositions.

How many DOPC, Chol and SM molecules does a single nanodomain contain? Since nothing was *a priori* known about the distribution of individual lipids between the nanodomains and the remaining bilayer, two extreme situations were assumed: 1) Lipids are distributed equally between the nanodomains and the rest of the bilayer; 2) Lipid molecules of a given type are exclusively localized inside the nanodomains. The calculation of the number of lipid molecules in a domain is straightforward in both cases:

Situation 1: $N_{\text{inside}}^{i} = \pi R_D^2 Arx^i / a_0$ (5)

Situation 2: $N^i_{\text{inside}} = \pi R_D^2 x^i / a_0$ (6)

N^i_{inside} is the number of lipid molecules of type i inside a domain, R_D is the domain radius, *Ar* the total area occupied by the domains, x^i the molar ratio of the i-th lipid and, finally, a_0 the average lipid headgroup area. In this rough estimate, a constant value of a_0 was assumed for each different type of lipid.

Analysis of z-scan FCS data. Z-scan FCS is a technique by which absolute diffusion coefficients *D* in a planar bilayer can be obtained. This is achieved by measuring fluorescence autocorrelation functions (ACF) at well-defined positions along the optical axis of the microscope (the *z* axis). The acquired ACFs are fitted to a model that assumes Brownian diffusion of a dye in a two-dimensional surface and transition of the dye to the triplet state[5]

$$G(\tau) = 1 + \frac{1}{PN}\frac{1}{1+(\tau/\tau_D)}\frac{1-T+T\exp(-\tau/\tau_T)}{1-T} \qquad (7)$$

Here τ is a so-called lag-time, *PN* is the particle number, τ_D the dye diffusion time, *T* the fraction of the dye in the triplet state and τ_T the lifetime of the triplet state. Due to the Gaussian beam profile, τ_{2D} and *PN* values follow a parabolic dependence with Δz, which allows for the determination of *D* according to[6]

$$\tau_{2D} = \frac{w_0^2}{4D}\left(1 + \frac{\lambda^2 \Delta z^2}{\pi^2 n^2 w_0^4}\right) \qquad (8)$$

where *n* is the refractive index, λ is the excitation wavelength and Δz the distance between the actual sample position and a reference position z_0.

t-test confirms the significance of the desrease in the diffusion coefficient due to the presence of nanodomains. To test the significance of the drop in the diffusion coefficient after

nanodomains were formed a so-called t-test was performed (Tables SI2 and SI3). This test can be used to determine if two sets of data are significantly different from each other. It provides a parameter called *p*-value, which determines the significance of the drop in *D*. *p*-values lower than 0.1 or 0.05, respectively, determine a significant change in *D*. In Tables SI2 and SI3 *D* in a homogeneous bilayer (no nanodomains) is compared with *D* in a heterogeneous bilayer (with nanodomains). For instance, the lipid mixture DOPC/Chol/SM (100/0/0) is compared with the mixtures DOPC/Chol/SM (90,88,85/0/10,12,15).

Table SI2: Statistical test (t-test) of the significance of the drop in the diffusion coefficient that was caused by the formation of nanodomains in DOPC/SM (90-85/10-15) mixtures (compare with Fig. 4 in the manuscript). The drop is considered significant when the p-value obtained from the t-test is lower than 0.1.

DOPC	Chol	SM	nanodomains	probe	D[μm^2/s]	*p*-value	drop in *D*
100	0	0	NO	g-GM_1	9.4 ± 0.29	-	-
90	0	10	YES	g-GM_1	8.9 ± 0.43	0.0752	Significant
88	0	12	YES	g-GM_1	9.1 ± 0.30	0.1451	Insignificant
85	0	15	YES	g-GM_1	8.6 ± 0.23	0.0016	Significant
100	0	0	NO	DiD	10.0 ± 0.35	-	-
90	0	10	YES	DiD	9.9 ± 0.19	0.7073	Insignificant
88	0	12	YES	DiD	9.7 ± 0.32	0.2814	Insignificant
85	0	15	YES	DiD	9.4 ± 0.25	0.0697	Significant

Table SI3: Statistical test (t-test) of the significance of the drop in the diffusion coefficient that was caused by the formation of nanodomains in DOPC/Chol/SM (70-65/25/5-10) mixtures (compare with Fig. 4 in the manuscript). The drop is considered significant when the p-value obtained from the t-test is lower than 0.1.

DOPC	Chol	SM	nanodomains	probe	*D*[μm^2/s]	*p*-value	drop in *D*
100	25	0	NO	g-GM_1	9.1 ± 0.15	-	-
70	25	5	YES	g-GM_1	8.2 ± 0.42	0.0036	Significant
67	25	8	YES	g-GM_1	8.1 ± 0.26	0.0000	Significant
90	25	10	YES	g-GM_1	7.2 ± 0.25	0.0000	Significant
63	25	12	unstable comp.	g-GM_1	8.35 ± 0.63	0.0270	Significant
100	25	0	NO	DiD	9.2 ± 0.40	-	-
70	25	5	YES	DiD	9.3 ± 0.47	0.3533	Insignificant
67	25	8	YES	DiD	8.7 ± 0.12	0.0028	Significant
90	25	10	YES	DiD	8.3 ± 0.20	0.0002	Significant
63	25	12	unstable comp.	DiD	8.9 ± 0.16	0.3200	Insignificant

As follows from Tables SI2 and SI3, formation of nanodomains both in DOPC/SM and DOPC/Chol/SM mixtures leads except of one case to a significant decrease in *D* when using g-GM_1 probes. Diffusion of these probes is influenced by the presence of the nanodomains to a large extent because they are localized preferentially in the nanodomains. On the other hand, DiD probes are distributed equally between the nanodomains and the remaining bilayer, which hampers any impact of the nanodomains on the diffusion.

Synthesis of Rh-PEG-DSPE. 5 mg (1.7 μmole) DSPE-PEG2000 were dissolved in 100 μl of dried chloroform and mixed with a solution of rhodamine 101 (0.2 mg, 0.3 μmole) in 50 μl of dried chloroform, 10 μl (10 μl /100 μl of chloroform) of triethylamine and 1 mg (0.3 μmole) BOP. The reaction mixture was stirred for 1.5 h at 4 °C and kept at 0 °C overnight. The reaction mixture was evaporated and dissolved in 100 μl of chloroform. Rh-PEG-DSPE was separated by column chromatography (Silica gel 60, Merck) in chloroform : methanol : H_2O, 65:25:1 (v/v). After evaporation, 0.42 mg (0.13 μmol, 38 %) of Rh-DSPE-PEG2000 was obtained as a red powder.

Chemicals. 1,2-dioleoyl-*sn*-glycero-3-phosphocholine (DOPC), GM_1 ganglioside (Ovine brain sodium salt), N-stearoyl-D-erythro-sphingosylphosphorylcholine (SM), cholesterol, 1,2-

dipalmitoyl-*sn*-glycero-3-phosphoethanolamine-N-(cap biotinyl) (biotinyl-PE) and 1,2-distearoyl-*sn*-glycero-3-phosphoethanolamine-N-poly(ethyleneglycol)2000 labelled in the headgroup region by carboxyfluorescein (CF-PEG-DSPE) were purchased from Avanti Polar Lipids (Alabaster, AL, U.S.A.) and used without further purification. DiIC18(5)-DS (DiD) was purchased from Invitrogen (Carlsbad, CA) whereas Streptavidin and biotin labeled bovine serum albumin (biotin-BSA) were purchased from Sigma (St. Louis, MO). DOPE labeled in the headgroup region by Atto-488 (Atto-488-DOPE) or by Atto-633 (Atto-633-DOPE), respectively, was bought from ATTO-TEC GmbH. Synthesis of FL-BODIPY-GM_1 (g-GM_1)[7] and of 564/570-BODIPY-GM_1 (r-GM_1)[8] has already been described previously. The synthesis of 1,2-distearoyl-*sn*-glycero-3-phosphoethanolamine-N-poly(ethyleneglycol)2000 labeled in the headgroup region by Rhodamine-101 (Rh-PEG-DSPE) is described in SI. The following compounds were used for the synthesis: 1,2-distearoyl-*sn*-glycero-3-phosphoethanolamine-N-[amino(Polyethyleneglycol)2000]ammonium salt (DSPE-PEG2000, purchased from Avanti Polar Lipids), Rhodamine 101 perchlorate (Lambda Physik, Germany), triethylamine (Fluka), benzotriazol-1-yloxytris-(dimethylamino)-phosphonium hexafluorophosphate (BOP, purchased from Fluka) and silica gel 60 (40-63 μm, purchased from Merck GmbH). All solvents used here were of at least analytical grade.

References

1. Johansson, L. B.-Å., Engström, S. & Lindberg, M. Electronic-Energy Transfer in Anisotropic Systems. 3. Monte-Carlo Simulations of Energy Migration in Membranes. *J. Chem. Phys.* **96,** 3844–3856 (1992).
2. Marushchak, D., Gretskaya, N., Mikhalyov, I. & Johansson, L. B.-Å. Self-aggregation - an intrinsic property of G(M1) in lipid bilayers. *Mol. Membr. Biol.* **24,** 102–112 (2007).
3. Sezgin, E. *et al.* Partitioning, diffusion, and ligand binding of raft lipid analogs in model and cellular plasma membranes. *Biochim. Biophys. Acta - Biomembr.* **1818,** 1777–1784 (2012).
4. Šachl, R., Johansson, L. B.-Å. & Hof, M. Förster resonance energy transfer (FRET) between heterogeneously distributed probes: Application to lipid nanodomains and pores.

Int. J. Mol. Sci. **13,** 16141–16156 (2012).

5. Widengren, J., Mets, Ü. & Rigler, R. Fluorescence Correlation Spectroscopy of Triplet States in Solution: A Theoretical and Experimental Study. *J.Phys.Chem.* **99,** 13368–13379 (1995).
6. Benda, A. *et al.* How to determine diffusion coefficients in planar phospholipid systems by confocal fluorescence correlation spectroscopy. *Langmuir* **19,** 4120–4126 (2003).
7. Bergström, F. *et al.* Dimers of dipyrrometheneboron difluoride (BODIPY) with light spectroscopic applications in chemistry and biology. *J. Am. Chem. Soc.* **124,** 196–204 (2002).
8. Marushchak, D., Gretskaya, N., Mikhalyov, I. & Johansson, L. B.-Å. Self-aggregation - an intrinsic property of G(M1) in lipid bilayers. *Mol. Membr. Biol.* **24,** 102–112 (2007).
